# The role of ethics in science: a systematic literature review from the first wave of COVID-19

**DOI:** 10.1007/s12553-021-00570-6

**Published:** 2021-06-03

**Authors:** Alessia Maccaro, Davide Piaggio, Silvio Pagliara, Leandro Pecchia

**Affiliations:** 1 School of Engineering, University of Warwick, Coventry, CV47AL UK; 2 Institute of Advanced Study, University of Warwick, Coventry, CV47AL UK; 3 European Alliance of Medical and Biological Engineering and Science (EAMBES), Leuven, Belgium; 4 IUPESM, York, UK

**Keywords:** Ethics, Systematic literature review, Covid-19, First wave, Public health

## Abstract

This paper proposes a systematic literature review on ethics and CoviD-19, aiming to understand the impact and the perception of the pandemic during the first wave (January-June 2020) and the consequences one year later. PubMed was systematically searched up May 2020 to identify studies that took into consideration various ethical issues that have been arising from the Covid-19 outbreak. The eligibility of the papers was determined by two authors, who screened the results mediated by a third author. In order to facilitate the screening, the titles were divided into five sub-thematic macro-areas, namely *allocation*, *policy*, *specialist*, *clinical trials*, and *technology* and, when possible, per geographical area. Specifically, a posteriori, we decided to focus on the papers referring to *policies* and *technology*, as they highlighted ethical issues that are not overused and worthy of particular attention. Thus, 38 studies out of 233 met our inclusion criteria and were fully analysed. Accordingly, this review touches on themes such as fairness, equity, transparency of information, the duty of care, racial disparities, the marginalisation of the poor, and privacy and ethical concerns. Overall, it was found that despite the increased awareness of interdisciplinarity and the essential reference to ethics, many scientific articles use it with little competence, considering it only a "humanitarian" enrichment. In fact, as we understand, reflecting a year after the outbreak of the pandemic, although Covid-19 is leading scientists to increasingly recognise the importance of ethical issues, there is still a lot of confusion that could be helped by establishing international guidelines to act as a moral compass in times of crisis.

**Supplementary information:**

The online version contains supplementary material available at 10.1007/s12553-021-00570-6.

## Background

The spread of COVID-19 begot what the World Health Organisation (WHO) defined a “pandemic” [1], an emergency condition that has often been compared to other dramatic events in history (e.g., the Spanish flu in 1918). However, on closer inspection, COVID-19 can be regarded as an unprecedented event with its own specificity.

During the first months of the pandemic, the numerous emerging issues were deeply interwoven with ethics, as it is unanimously recognized by the works of politics, medicine, and science. The interdisciplinarity of such works and their continuous and necessary reference to ethics reinforce the belief that bioethics, i.e., a "bridge" between different fields of knowledge, looks more and more "towards the future" [2]. In fact, in the first few months, the contributions of National Ethics Committees, International Organizations, National Bodies and Professional Association to this topic [3] were numerous, very rapid, and accompanied a very heated debate on ethical implications between the population and experts from various backgrounds. Nonetheless, the continuous reference to the word “ethics” in the first publications after the declaration of COVID-19 pandemic [4–7] highlighted the need to clarify the most relevant ethical problems related to the scientific community. Hence, it was decided to focus on and deepen the subjects that aroused the interest of specialists the most. Specifically, existing works of reconstruction and/or comparison among national and international documents on the relationship between ethics and COVID-19 were not taken into consideration, to avoid delving into or discoursing about already exhaustively discussed topics.

Consequently, a systematic literature review was opted for. Such review was conducted by an interdisciplinary working group, comprising biomedical engineers and bioethicists, following a multidisciplinary approach, which overcomes the outdated Cartesian model of the separation of knowledge into “silos” of disciplines [8, 9]. To facilitate the screening process, the papers were grouped by macro areas that were pinpointed through a thematic analysis [10], as explained in the methods. In particular, it was decided to analyse only the themes related to policy, technology, resource allocation, and low- and middle-income countries (LMICs) as they fall within our competences and seem to underline relevant ethical questions, which are not always adequately or exhaustively discussed. However, the theme of resource allocation was excluded a priori, because it is a trite topic, currently “abused”, on which anyone expresses their opinion independently from their level of expertise and knowledge. Moreover, many papers, which had a reference to ethics in the title, but that, in fact, did not deal with analysing the ethical implications of the investigated issues, were excluded. In fact, the ultimate purpose of this work was to understand the role that scientists recognize in ethics and the problems related to it: is it seen as a “humanitarian” addition to technical issues or as a structural element and perspective from which to analyse specialistic issues?

Therefore, this paper reports a collection of the most relevant and less conventional ethics challenges related to COVID-19 published in peer-reviewed journals indexed in PubMed, analysed through our multidisciplinary lens. These ethical issues, which emerged during the first wave of the pandemic, were then rediscussed a year later, in order to assess whether the first bioethical perspectives related to COVID-19 were biased by the close succession of the events being analysed, or they were detached enough, and the raised issues remain current a year on.

## Methods

### Systematic literature review

Given the large number of papers and documents that have been and are currently being published since the start of the pandemic, we decided to conduct a systematic literature review. PubMed was selected as the only database to identify all the contributions published from 01/01/2020 up to 19/05/2020 responding to the topics of COVID-19 and ethics. The search string was constructed with the following terms combined with the Boolean operators AND/OR: “ethics”, “ethical”, “bioethics”, “COVID”, “sars cov 2”, “coronavirus” ((ethics OR ethical OR bioethics) AND (COVID OR sars cov 2 OR coronavirus)). We judged eligible only the papers with full text available and in English. In order to facilitate the clustering and further screening of the retrieved articles, they were divided into the most recurring themes and per geographical area. To this regard, the countries were grouped according to the following macro groups, based on the retrieved papers: North America, Europe, LMICs (e.g., Tunisia), Asia, and Israel. Particular attention was dedicated to the contributions regarding LMICs. Finally, only the paper pertaining to our field of expertise, i.e., technologies and policy, made it through the final selection. During this process, two authors independently screened all the titles and abstracts for eligibility. Full texts were considered if the selection was unclear. A third author reviewed and checked the results of the screening search. Any discrepancy was resolved by discussion among all the authors. All the relevant papers were analysed, summarised and coded to facilitate the reading.

## Results

### Systematic literature review

**Fig. 1:**
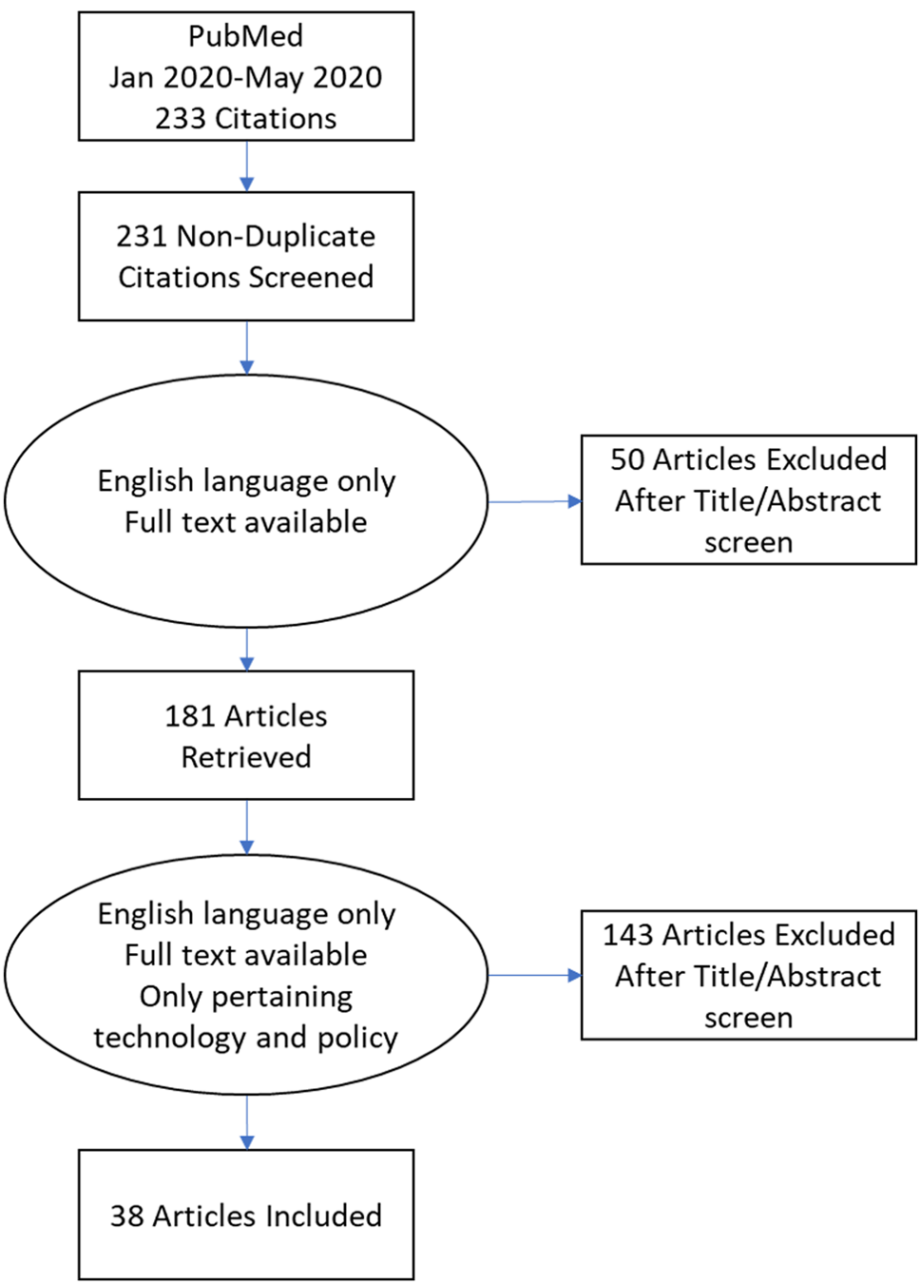
The flowchart of the systematic literature review

**Fig. 2:**
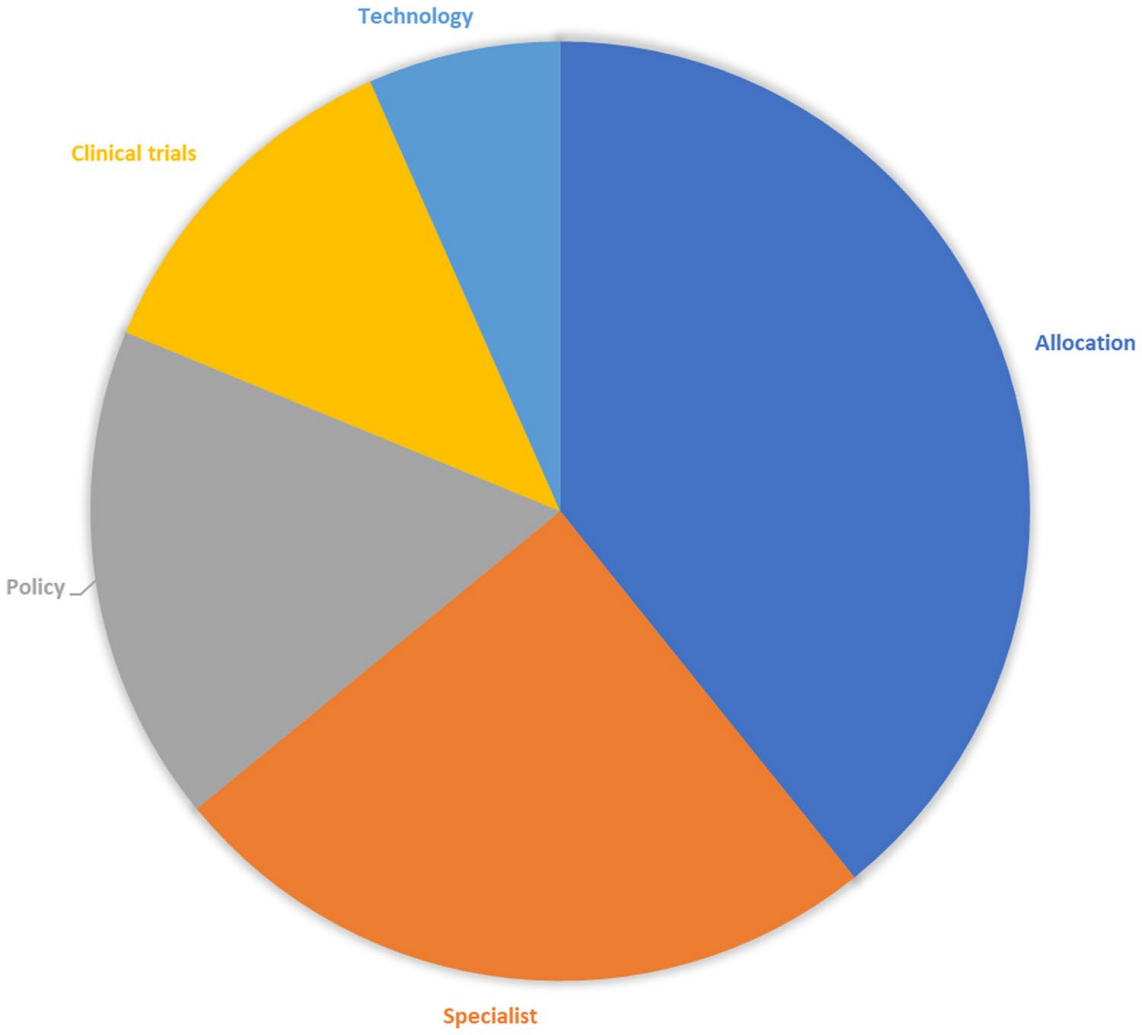
The distribution of the articles in 5 macro-areas

**Fig. 3:**
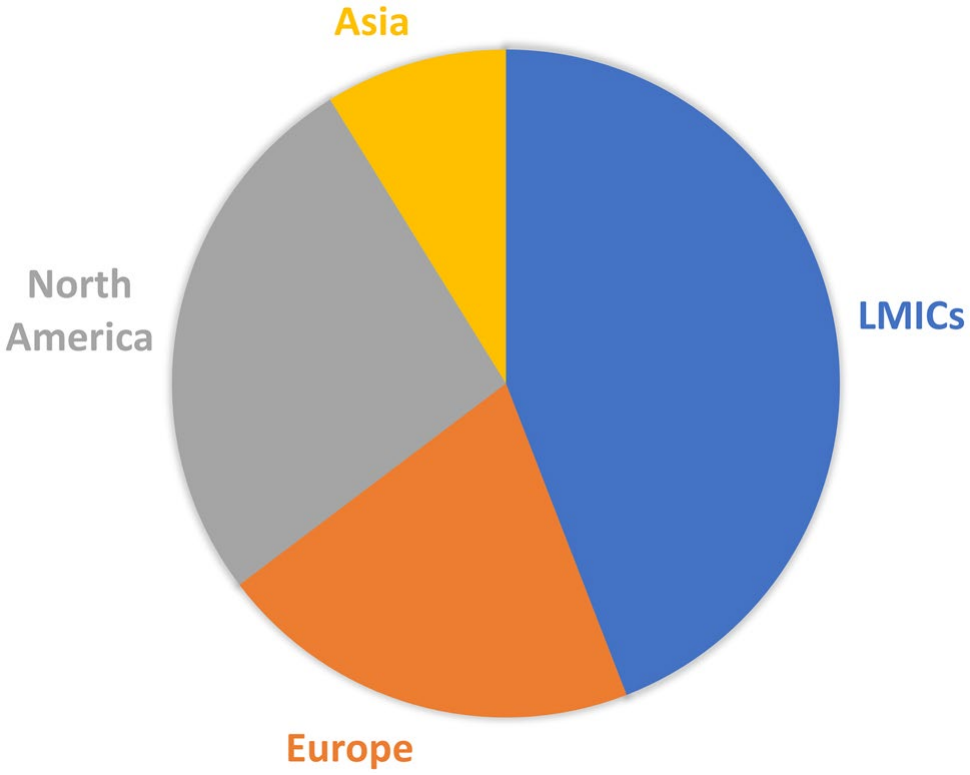
The distribution of the articles per geographical area

Figure 1 shows the electronic database search and the selection process. Only 38 papers out of the initial 233 resulted eligible to be included in our study, which focuses on technology and policy. Figure 2 shows the division in macro groups and Fig. 3 the division per geographical area, when applicable. Online Resource 1 presents all the selected papers organised by recurring themes.

The 5 pinpointed macro groups of specific works on ethics and COVID-19 were: Policy, Resource allocation, Specialists, Clinical trials, and Technology. The theme of policies, i.e., of the public responses to the crisis, is the one that piques the scientists’ interests the most, according to our review. In this regard, as already mentioned, our review is not exhaustive and does not aim at including all the political guidelines of the various countries, because its hermeneutical horizon [11] is the point of view of science. Notwithstanding, it was possible to identify a series of specific works on different countries, which allowed comparisons between different areas of the world: LMICs, Asia, Europe, and North America.

The theme of resource allocation is also extremely well-liked. In fact, the first wave of the pandemic caused a scarcity of resources, globally, without any distinction: from personal protective equipment (PPE) to medical devices (MDs) (e.g., ventilators, respirators), beds, drugs for COVID-19 patients or patients suffering from other pathologies, health personnel, and COVID-19 tests. Most specifically, this situation of emergency abruptly showed the lack of competent ad-hoc bodies. This begot extremely heterogeneous approaches to ethics by different individuals, regardless their competence in ethics.

Among the retrieved publications, there were also numerous articles on ethics and COVID-19 written by specialists from various medical fields (e.g., geriatrics, psychiatry, surgery, oncology, and dentistry). Other publication focuses on all healthcare workers (HCWs), such as nurses and midwives, who experienced profound difficulties in this particular historical moment, the common denominator being allocation problems.

A significant number of works was related to clinical trials, in particular concerning vaccines and drugs, dealing with detecting the widespread condition of researchers who had to readdress the scopes of their research, always respecting high ethical standards and safeguarding the testers.

Finally, the last area is that of technology for COVID-19 (e.g., eHealth) and all the related ethical issues.

## Discussions

### The duality of trust: on the trustworthiness of governments and public trust

Most of the articles included the topic of *infodemic*, i.e., “an over-abundance of information – some accurate and some not – that makes it hard for people to find trustworthy sources and reliable guidance when they need it” [12]. Misinformation circulating through global digital social networks in the first months of this pandemic was focused on trust in governments and policy makers [13–15], questioning the principles of legitimacy and responsibility related to information verification and sharing.

But who was the source of such disinformation?

Larson [15] claimed that it is was the governments who repressed information hoping to calm anxious publics, as it happened in Iran [15, 16] or in China, where healthcare workers, who told the truth about the spread of the virus and the scarcity of resources, were looked at as “whistle-blowers” and forced to withdraw their declarations [17]. Moreover, some governments deliberately released supposedly reassuring misinformation, risking undermining their own credibility and their abilities to help people counter real health threats. According to Limaye et al. [13], there is a need for joint action between government agencies and social media companies for fact-check and even removal of false or outdated information. On the other hand, according to Bastani and Bahrami [16], there is the need of an active and effective presence of health professionals and authorities on social media, due to the poor legal supervision of online content.

Low-quality research contributed to this misinformation, too. In fact, the high demand for information caused an acceleration in reporting scientific results, with many journals publishing without any peer-review and offering open-access to everyone. A proxy for the high number of low-quality research on COVID-19 is also the unprecedented high number of retracted papers. To this regard, we searched for papers on COVID-19 or SARS-Cov-2 and the previous epidemics/pandemics (i.e., avian flu, swine flu, MERS) both on the Retraction Watch Database and OvidSP. As a result, as of May 2021, 124 out of 264,530 papers on COVID-19 (4.68 retractions per 10,000) were retracted, compared to 1.16 per 10,000 papers concerning the previous pandemics/epidemics.

Apart from being unethical, reporting poor quality outcomes is a kind of research misconduct [18] and inaccuracy. This could also have other consequences, for example exacerbating stigma and discrimination against particular populations [19]. In fact, Chowkwanyun and Reed [20], analysing the information circulated in the media in Wisconsin and Michigan on the high percentage of black people affected by Coronavirus, argued that there is always the need for contextualisation unless we want to foster harmful myths and misunderstanding, which undermine the goal of eliminating health inequities. The fear of stigmatization towards specific groups of people is likely to worsen if they are individuals. In this case, the ethical dilemma is the balance between personal and collective interests.

In hindsight, the public health measures that have been implemented can be of different types. Sulmasy and Veatch [21] identifies four of them:

1. Contact tracing through the self-reporting of recent close interactions by people known to be infected with COVID-19. In this regard, Luo et al. [22] reported the example of an online questionnaire that circulated in China as an internet approach in COVID-19 participatory surveillance.
2. More "draconian" health measures: new surveillance technologies that employ facial recognition, security cameras, and phone GPS monitoring could attempt to identify everyone who spent at least fifteen minutes within six feet of every infected individual. Each contact could be forcibly quarantined. In this regard, [23] underlined that apps could also be used as a preventive approach. However, as Vokinger et al. [24] affirmed, trustworthiness and integrity of contact tracing apps should be assessed with a framework. A review of the tracing apps for the management of COVID-19 can be found in [25], where the authors underlined that among the advantages there were the increase of personal freedom, of personal feelings of safety, and the improvement of the management of the quarantine. However, governments should implement policies to outline requirements for these apps and should safeguard privacy, access, transparency, the protection and use of these data [24, 25]. In particular, Santow [26] underlined the need for a legal framework to regulate artificial intelligence and data sharing, as they can be the cause of discrimination and violation of human rights.
3. Voluntary contact tracing: it relies on the self-led contact tracing. However, it is not perfect because patients might not remember all their recent contacts.
4. The public naming of infected individuals: in this regard, Sulmasy and Veatch [21] described the case of the Prestigious University, in which the communication via email about the testing positive of a staff member raised a heated debate regarding the request to reveal the identity of the subject in order to maximize public health benefit and slow the spread of the virus. One of the authors argued that this could breach confidentiality and be harmful to the patient's privacy, who is free to decide whether to make a voluntary disclosure. However, another author believed that the confidentiality breach is morally mandatory to decrease the risk of contagion for other members of the University, because the duty towards the community has priority over the right of confidentiality. This is also discussed by Persad and Emanuel [27], who reported the proposal of some states (i.e., Chile, Germany and the UK) to implement “certifications of immunity” or “immunity passports” for those who had COVID-19 or who will have received the vaccine in the future. In line with the principle of the “least restrictive alternative” to achieve public health objectives, the author believe that this tracking measure is not unethical and cannot be compared to the yellow star that the Nazis forced the Jews to wear, because it is not a form of discrimination. However, it needs careful implementation and scientific support to be ethical in practice.

Another category of individuals who place the interests of society before their own is that of frontline healthcare workers (HCWs) who are forced to work in precarious conditions at their personal risk [28]. However, for them there is also another ethical dilemma: that of protecting, together with one's own health, also one's family and loved ones, which often clashes with the duty to treat patients, sometimes in the absence of PPE [29, 30]. According to McConnell [31], there are several factors that adjust the burden of protecting one's family. The authors argued that the moral demands of "Samaritanism" (i.e., one should go out of one's way to help someone else, if it entails a little cost to oneself) do not imply that HCWs take on the risks and burdens associated with treating COVID-19 to save several lives. Likewise, Thomas et al. [30] denounced the low quantity and quality of PPE and the inadequacy of the related guidance issued by Public Health England. Moreover, he appealed to the precautionary principle and praised the ethical framework of Beauchamp and Childress [32] that encourages to counterbalance beneficence with non-maleficence. Always according to Thomas JP, political leaders have the moral duty to be open and honest, when informing all frontline HCWs of their own personal risks in caring for COVID-19 patients.

Nonetheless, as in all the aspects of life, economical evaluations cannot be excluded. Hilsenrath [33] presented a very sensitive issue, that of confronting the medical duty to save lives and the reasoning of economists, who invite people to make decisions on appropriate costs. The author lucidly underlined that although the issue is often bypassed by political leaders, especially Americans, it should be faced by considering an ethical balance, certainly painful, between the ethical and economic damage that countries are experiencing in this historical moment.

Overall, what is required from governments is trust. People place their trust, their lives, health, and economic situation in a sensible and transparent decision-making of governments. Therefore, as Thomas et al. claimed, "it is a reasonable expectation to hold our modern governments to the corresponding standards of our modern health professionals: specifically, transparency in decision-making and the duty of candidate" [30]. Similarly, Lewnard and Lo [34] sustained that policymakers maintain the public's trust through the use of evidence-based interventions and fully transparent fact-based communication. To give an example of this, the author also reported on mathematical modelling of the viral transmission under different scenarios to generate evidence of the efficacy of social distancing interventions.

On the other hand, Chaari and Golubnitschaja [35], supporter of 3P (predict, prevent and personalise) medicine, proposed “real-time” monitoring based on randomized laboratory tests as a source of evidence. However, this clashed with the problem of the lack of tests and the discrepancy between officially recorded and real infection cases. In fact, this could lead to incorrect political decisions heavily influencing the future of a country (e.g., a long-term economic recession due to over-protection of the population, or a post-containment pandemic rebound due to an under-protection of the population). Furthermore, all the measures required from governments (i.e., testing, screening, contact tracing, social distancing, travel restriction) must be as inclusive as possible in particular with vulnerable communities (i.e., homeless, those without insurance or employment, disabled, immigrant, prisoners) [14, 34]. This also means being aware that the use of technology to combat the spread of COVID-19 could exacerbate racial, socioeconomic and geographic disparities for populations that lack access to reliable internet access, devices or technological literacy [36]. Moreover, during the pandemic, the benefits of technologies that allow relationships beyond social distancing have increasingly been experienced and could be a valid assistance tool for the elderly [37] and favour the communication of hospitalised patients with their families [36]. This response should also involve millennials and Generation Z more [38], as, despite the negative stereotypes that circulate around them, they could offer valuable help to overcome this crisis.

Certainly, the use of technology, if regulated, can be of help to scientists. Indeed, O'Reilly-Shah et al. [39] highlighted the shortcomings in the US healthcare IT infrastructures, underlining the importance of the interoperability of healthcare data, which should refrain from the proprietary control of vendors and be accessible to healthcare providers, especially in times of crisis.

More generally, it could be said that technology and data sharing are particularly important for disseminating knowledge worldwide. Momtazmanesh et al. [40] sustained that sharing and solidarity are at the base of an indispensable international collaboration to fight the current and future pandemics. The pandemic has increasingly shown the need for an international ethical–political coordination framework [23], aiming at reducing disparities. In this regard, under the category of *policy*, many contributions regarded LMICs. One of the recurrent themes was the stress on already overburdened and underfunded public healthcare systems in Africa, India, and Latin America [41–45]. In a more general comparison between the North and the South of the world, Schuklenk et al. [43] affirmed that there is a significant difference in the number of available intensive care unit beds per population and that the access to cures is often wealth-based, as many hospitals in the South are private. However, Krishna [42] stated that COVID-19 is just another drop in an already full vase, as the Indian healthcare system is already plagued by internal issues (e.g., no access to medicines, vested political interests etc.). All in all, the policies against COVID-19 are, once again, putting the poorest countries and people at risk [43, 46]. In India, for example, the poor were confined in ghettos without a proper social security net and their conditions were exacerbated by the lockdown [46]. Other approaches used in LMICs to fight COVID-19 include the use of fear appeals to regulate people’s behaviours [47]. However, it can be argued whether this technique is ethical and acceptable or not. On the other hand, Kapata et al. [48] positively affirmed that Africa was readier than for the previous epidemic outbreaks.

Other diseases, such as vector-borne and non-communicable diseases, and community-acquired infections are often included among other stressors [41, 42]. Krishna [42] also affirmed that notwithstanding this, COVID-19 obtained much more funding compared to other existing deadly diseases (e.g., diarrhoea).

Another element contributing to exacerbating the situation is the lack of ethics committees in the hospitals to regulate and ease the work of healthcare operators. In fact, the latter do not only have to face very difficult choices in an environment dominated by material scarcity and public distrust [41], but they also have to work in precarious conditions and their striking for this matter could be seen as patient abandonment [43]. With regard to this, Gopichandran and Subramaniam [44] recalled the *reciprocity principle*, according to which the state should protect the interests of the healthcare workers, who risk their lives to care for those who are infected.

Some authors [16, 44, 45, 49] agreed on the need for equity and clarity in the way governments inform citizens, not to undermine their trust. Most importantly, it was unanimously stated that policies for COVID-19 should always be adapted to different contexts, above all the ones related to minorities, in the respect of traditional beliefs [50], and to avoid exacerbations of pre-existing gaps between the rich and the poor [46]. To this purpose, self-determination is key [51]: in fact, LMICs should independently shape their response, relying on international partners in a critical manner. For instance, LMICs should avoid the unconditional acceptance of measures adopted in high-income countries that would result inappropriate in resource limited settings [52–54].

Overall, the number and significance of the ethical problems that emerged, which makes us understand how ethics should be increasingly involved in guiding political and scientific legal reasoning, lead us to disagree with Stoeklé and Hervé [55], who, by separating political discussions, scientific knowledge and ethics writes: "now really isn't the time for ethical reflections" and "ethics is only really useful if you have the time, and right now, time is exactly what we do not have."

## Conclusions

This paper presented a systematic overview of scientific papers investigating ethical issues during the first wave of this pandemic. The papers highlighted some recurrent themes, namely the allocation of scarce resources, infodemic, HCWs’ duty to treat versus personal protection, privacy, and the safeguard of minorities.

Reviewing such themes, a year after the outbreak of COVID-19, highlights several facts.

The infodemic has been as devastating as the virus. While it could have been acceptable in the first months of this pandemic, it is somehow surprising that after three waves of COVID-19, there is still confusion around trustworthy sources and reliable guidance. A year after, there is still misinformation spread through digital social networks, although the object of the discussion has changed, focusing now on vaccination safety and scarcity, rather than MDs and PPE. The same doubts raised by Larson [15], Limaye et al. [13] and Bastani and Bahrami [16] in March 2020 on the intentional repression of information perpetuated by governments for calming anxious publics during the first wave, have been recently repeated in regard to the surge of COVID-19 that affected India since March 2021.

After more than 12 months from the first wave, the ethical concerns on the stigma and discrimination against particular populations is still relevant in many ways. For instance, several authors hypothesize a causal link between COVID-19 and the surge of violent acts towards Asians living in the USA [56, 57]. On the global scale, this discrimination will certainly not be mitigated by the delay in vaccinating populations in LMICs.

Moreover, the ethical concerns arising from balancing the need for track and trace with the risks for privacy, seem to be still unresolved. After a year and half into the pandemic, the same scepticism has crossed many COVID-19 waves resulting in several Apps being abandoned for more traditional phone-based methods. Similarly, the envisioned “real-time” monitoring based on randomized laboratory tests is still far from being possible and could still lead to incorrect political decisions heavily influencing the future of a country. One year on, in fact, although this risk has lowered, globally, it still is critical for some LMICs, where several challenges hinder COVID-19 testing [58].

Concerns on immunity certifications and pass, are yet far from being resolved, although the attention has now shifted towards the vaccines.

Concerning HCWs, the memory of recognising their efforts by cheering and clapping from the windows is far.

The scarcity of PPE and MDs seems to be overcome now, in many high-income countries, also thanks to the massive effort of United Nation agencies lead by the World Health Organization. Yet, the problem is far from being solved without structural interventions, as demonstrated by the surge in cases in India.

As regards the ethical issues on the appropriateness of economical evaluations and the transparency of political decisions, raised by authors during the first months of this pandemic, there is a need for more time before one can make unbiased reflections.

Inclusiveness seems to be one of the forgotten principles during this pandemic. While this issue was raised from the very start, the question remains open for several groups of populations, which have been not prioritised during the first wave, such as children, poor, unstable workers, chronic patients, and single parents.

Overall, this review identified a number of papers referring to ethics in their title, which in the end presented little or no contribution to the ongoing discussion on ethical issues arising from COVID-19. While the attention of many scholars to this important topic is remarkable, we conclude this review with the doubt that ethics is still considered as a tick-box exercise by many, when not a “humanitarian embellishment” of their technical work.

Conversely, ethics should be deeply interwoven with science, theoretically and methodologically. Besides, helping the evaluation of the arising issues, ethics could help scientists ponder on the risk–benefit balance of their publications, and also on the final purpose of their work, i.e., the progress of humanity. The unprecedented number of retracted papers suggests that during this pandemic the race to participate to the infodemic overcame the sense of responsibility, which should have imposed, in many cases, a responsible silence [59].

## Supplementary information

Below is the link to the electronic supplementary material.

Supplementary file1 (DOCX 30 KB)

## Data Availability

The results of the systematic literature search are available by contacting the authors upon reasonable request.

## Abbreviations

WHO: World health organization
LMICs: Low- and middle-income countries
PPE: Personal protective equipment
MDs: Medical devices
HCWs: Healthcare workers
