# Supplementary material for: The role of ethics in science: a systematic literature review from the first wave of COVID-19
Source: Health Technol (Berl). 2021 Jun 3;11(5):1063–71. doi: 10.1007/s12553-021-00570-6 (PMC8175060; doi:10.1007/s12553-021-00570-6)
Supplement: Supplementary file 1 — Supplementary file1 (DOCX 30 KB) [file 12553_2021_570_MOESM1_ESM.docx]

# Title: The role of ethics in science. A systematic literature review from the first wave of COVID-19

Journal: Health and Technology

Alessia Maccaro^1*^, Davide Piaggio^1^, Silvio Pagliara^1^, Concetta Dodaro^2^, Leandro Pecchia^1^

^1^School of Engineering, University of Warwick, CV47AL, Coventry, United Kingdom

^2^Advanced Biomedical Sciences, University of Naples Federico II, Naples, Italy

*Corresponding author: Alessia.maccaro@warwick.ac.uk

Online Resource 1. A table summarising and grouping all the papers included in the systematic literature review.

| **ID** | **Theme** | **Keywords** |
| --- | --- | --- |
| [17] | Policy | - Infodemic - Transparency of communication from healthcare workers - General public health goal as a measure of ethicality |
| [55] | Policy | - Ethics as a post-reflection - Healthcare operators’ own ethical values as a measure for their action in times of emergency |
| [16] | Policy | - Infodemic - Social media as a source of misinformation - Health professionals and authorities on social media and the improvement of public health literacy as a solution |
| [20] | Policy | - Racial disparities - Importance of a contextualised analysis, to avoid the spread of wrong information - Transparency of information |
| [18] | Policy | - Infodemic - Transparency of communication - Reliability of communication - Use of social media to communicate - Empowerment of low-resource local communities |
| [19] | Policy | - Infodemic - Transparency of communication - Reliability of communication - Publication ethics to be respected |
| [27] | Policy | - Certifications of immunity - Fairness VS individual freedom and improvement of public health - Protection of liberty and autonomy through the “least restrictive alternative” - Avoidance of stigma and inequality |
| [28] | Policy | - Healthcare operators are facing very difficult choices and work in precarious conditions - Best interests of individuals < bests interest of society during a pandemic |
| [36] | Policy | - Adoption of family-centric procedures (e.g., videocalls) - Reliance on technology may exacerbate disparities |
| [29] | Policy | - Healthcare operators are facing very difficult choices and work in precarious conditions - Fairness, harm principle, use of the least coercive and restrictive means, proportionality, and flexibility |
| [21] | Policy | - Infodemic - Safeguard of privacy VS public naming of infected individuals - Our duty to the community must be balanced against the right of confidentiality. |
| [33] | Policy | - Ethical balance to mitigate health and economic damage - Economic downturn as an opportunity for regeneration |
| [15] | Policy | - Infodemic - Transparency of information - Reliability of information - Increase of trust |
| [34] | Policy | - Social distancing intervention - Mathematical modelling of transmission - Marginalisation and exacerbation of the condition of the poor - Infodemic |
| [31] | Policy | - Healthcare operators are facing very difficult choices and work in precarious conditions - Duty to work and duty to protect the family for HCWs - Incentives for staff in frontlines |
| [40] | Policy | - International collaboration and solidarity during pandemic outbreaks |
| [14] | Policy | - Adaptation and contextualisation of policies to different contexts - Infodemic - Equality for access to healthcare, free testing, sick leave expenses paid by governments |
| [35] | Policy | - Mathematical modelling of transmission - 3p medicine: predictive, preventive and personalized |
| [30] | Policy | - PPE in the UK - Precautionary principle - The moral duty of government to be open, honest, transparent during the decision making - Duty of candour |
| [13] | Policy | - Infodemic - Partnership government and social media giants to supervise information |
| [26] | Policy | - A legal framework to protect from AI - Surveillance - Discrimination - Human rights |
| [41] | Policy (LMICS) | - Stress on the overburdened and underfunded public healthcare system - Other diseases are still a burden and cannot be neglected - Denial from political leaders and slow response - Healthcare operators are facing very difficult choices |
| [50] | Policy (LMICS) | - Adaptation and contextualisation of policies to different contexts - Respect for traditional beliefs |
| [42] | Policy (LMICS) | - Stress on the overburdened and underfunded public healthcare system - Other diseases are still a burden and cannot be neglected |
| [43] | Policy (LMICS) | - Stress on the overburdened and underfunded public healthcare system - Other diseases are still a burden and cannot be neglected - The poorest countries are put at risk by Covid-19 policies |
| [51] | Policy (LMICS) | - Adaptation and contextualisation of policies to different contexts |
| [44] | Policy (LMICS) | - Other diseases are still a burden and cannot be neglected - Healthcare operators are facing very difficult choices and work in precarious conditions - The poorest countries are put at risk by the Covid-19 policies - Stress on the overburdened and underfunded public healthcare system - Need for budget allocation to preventive and public health activities. - Need for improving public health surveillance system - Need for protecting frontline healthcare workers - Need for healthcare workers training. - Transparency of communication from healthcare workers |
| [46] | Policy (LMICS) | - Marginalisation and exacerbation of the condition of the poor - Ethics and equity to guide decisions - The global response should have been contextualised and tailored |
| [47] | Policy (LMICS) | - Fear appeals and use of threats - Moral examination of this technique |
| [48] | Policy (LMICS) | - Africa’s readiness for this outbreak - Summary of different kinds of interventions - Donations from different organisations |
| [45] | Policy (LMICS) | - Infodemic - Need for improving public health surveillance system - Privacy and ethical concerns - Stress on the overburdened and underfunded public healthcare system |
| [38] | Policy (US) | - Engaging all generations in the pandemic response will leverage intergenerational innovation and optimise the response to Covid-19 - Exhortation to engage young leaders in medicine and break down the traditional social hierarchy |
| [39] | Policy (US) | - Infodemic - Importance of data sharing to study Covid-19 - Importance of safeguarding the privacy of patients and healthcare workers |
| [22] | Technology | - Participatory surveillance (online questionnaire) |
| [24] | Technology | - A framework to assess the trustworthiness and integrity of contact tracing app and web apps. - Pros and cons of contact tracing apps in the management of the emergency due to covid19. |
| [25] | Technology | - Pros and cons of contact tracing apps in the management of the emergency due to covid19. |
| [37] | Technology | - Tool for older people and their families to improve health during social distancing - Anonymization of data |
| [23] | Technology | - Contact tracing - App as infection prevention approach (mathematical model) - Required advisory body for access, transparency, protection, use and sharing of personal data. |
